# Engaging Community Partners to Understand and Respond to Substance Use and Addiction Crisis Facing Families in Prince Albert, Saskatchewan

**DOI:** 10.1177/11782218221126881

**Published:** 2022-09-28

**Authors:** Geoffrey Maina, Marcella Ogenchuk, Jordan Sherstobitoff, Robert Bratvold, Barbara Robinson

**Affiliations:** 1 College of Nursing, University of Saskatchewan, Prince Albert, SK, Canada; 2 Saskatchewan Rivers Public School Division, Prince Albert, SK, Canada; 3 Saskatchewan Polytechnic, Prince Albert Campus, Prince Albert, SK, Canada

**Keywords:** Community-based research, substance use and addiction, the process of community engagement, families affected by addiction

## Abstract

Substance use is a persisting health care crisis that has led to residents’ addiction to diverse substances in Prince Albert, Saskatchewan. This public health issue affects not only those with a substance use disorder but also those within their circle of family and friends. This paper aims to outline the community engagement processes that we undertook to identify community priorities for addressing the substance use and addiction issues facing them. We began the community engagement using a patient-oriented research process, which led to the development of a grant application. Following the awarding of this grant application by the Saskatchewan Health Research Foundation and Saskatchewan Centre for Patient-Oriented Research, we conducted interviews with family members affected by addiction in the city. The study provided us with significant insight into the impacts of substance use disorders on family members. The importance of collaboration among people with lived experience, health care providers, and community partners helped us to identify our research questions. Community members also actively participated in the data collection, analysis, and presentation of the findings where priorities for the interventions were identified. The conversations we had because of the community’s engagement and participation in the research process enhanced our understanding of the realities of caring for people with substance use disorders and the importance of family involvement throughout the process. We also learned lessons regarding community engagement and participation in research on a stigmatizing and complex topic.

## Background

Prince Albert, Saskatchewan, is impacted by problematic substance use that contributes to significant social, legal, and health implications. The region has nearly double the per capita alcohol spending ($1249) for those 15 years and older compared to the provincial and national spending ($703 and $742, respectively).^1^ Although alcohol has been the main factor responsible for most cases brought to Community Mobilization Prince Albert (CMPA), a multi-agency team focused on crime prevention, CMPA also described that the community is experiencing methamphetamine crisis.^1,2^ Prince Albert and Region Alcohol Strategy report showed that 51% of crimes and 40% of reported violence were associated with alcohol and substance misuse.^1^ A study on alcohol use among grade 9 to 12 students in the Prince Albert region showed that initial use ranged from younger than 12 to 18 years old. The rate of high school students reporting using alcohol—32.2%—is 13% and 19% higher than the provincial and national rates respectively.^3^ About 67.9% of grade 10 students in the region reported binge drinking compared to the national average of 49.4%.^1^ Youth in this region also have a lower alcohol initiation age than provincial and national averages.^3,4^

Notably, Prince Albert also has one of the highest rates of injection drug use in the province. In 2015, 25.2% of the province’s clean needles were distributed in the region.^5^ These high rates prompted the City of Prince Albert to approve a future safe injection site that is yet to be carried out.^6^ Prince Albert’s HIV rate in 2019 was 56.4 people per 100 000, 3.4 times higher than the provincial rate and 8.2 times higher than the national rate.^7^ The most common risk factor for HIV is injection drug use, causing 67% of newly diagnosed infections.^7^ Finding effective ways to mitigate the negative consequences of substance use is crucial for Saskatchewan, where substance use is associated with a rise in sexually transmitted infections, family crises, and violence.^8,9^

Moreover, high alcohol consumption in the region significantly impacts law enforcement expenditures. For instance, between May 2009 and May 2012, the Prince Albert Police Services spent $2.5 million responding to unlawful activities associated with alcohol and substance use. In 2012 alone, the police service spent more than 1300 hours making more than 6000 arrests for public intoxication.^1^ High alcohol and substance use has also significantly increased health care service use. In 2017, the use of outpatient services for substance use and addiction was 24.4% higher than it had been in the previous year.^10^ About 18.2% of hospital admissions and 9.2% of all emergency room visits in Prince Albert at any given time are linked to substance misuse and addiction.^11^

In recognizing the magnitude to which the Prince Albert region is disproportionately impacted by substance use, the research team sought community input to understand the priorities for action through research. The objective of this paper is to describe the process through which the community identified priorities of concern and to describe their ensuing involvement in the research process phases. This process is broken down into 4 phases: (1) a knowledge exchange event, (2) the grant writing and award, (3) the execution of the study, and (4) knowledge translation activities. The results of this process will be reported in an outcome paper once the intervention is finalized.

### Phase 1: Knowledge exchange event

In this phase, we engaged the Prince Albert community to identify their priority issues that needed attention through a research project. We did not have a preconceived agenda at the time this event was hosted in 2017.^11^ Bringing the community together to focus on substance use was driven by the desire to provide an opportunity for them to voice the issues affecting them. It also allowed the university community to engage in outreach activities that could be mutually beneficial. During this event, dubbed “The change we want: A community engagement and knowledge exchange for substance use and addiction in Prince Albert,” our objective were to provide a space for community members to share their experiences about how problematic substance use was affecting their families and the community. The event provided a platform for community members and shareholders to share their experiences, identify gaps in providing care for people affected by addiction, and discuss how to respond to these identified needs. A significant gap identified as needing attention was responding to the impact of addiction on families.

Despite the known prevalence of substance use in Prince Albert, Saskatchewan, few resources target families affected by addiction. Most regional interventions are designed to address the physical, social, and mental health needs of people with problematic substance use.^12^ These include detoxification services, harm reduction programs, and interventions for mothers with addiction. The lack of attention to the impact of addiction on families and the lack of understanding of how families are affected by substance use disorders may explain the lack of investment to support them in the regional health care system.

In reviewing the literature on this topic, we noted that families, friends, and supporters are affected by addiction in distinctive ways.^12-15^ Copello et al note that addiction has a significant physical and psychological impact on families, which can be severe and long-lasting.^16^ Children of parents living with addiction often act as surrogates to their parents and work hard to restore social order and family interaction. In contrast, aging parents must maintain extended caregiver relationships with their adult children.^17^ Spouses often bear substantial financial and emotional burdens of supporting a partner with substance use disorder in the family.^18^ People often experience emotional hardships, including anger, frustration, depression, abandonment, anxiety, fear, embarrassment, and guilt, while caring for relatives with substance use disorder.^13,14,19^ Problematic substance use can also cause family instability through violence, divorce, disease risk, abuse, financial weakness, and the inability to provide for dependents’ needs.^14,15^

Family members may require professional help to manage the impact of living with a loved one with substance use disorder. However, stigma and the cost of treatment can exacerbate the family’s stressful experiences and constitute a barrier to accessing treatment.^16,20^ Moreover, self-care for family members is key to providing attention and appropriately supporting someone living with substance use disorder. To engage in self-care, family members have to prioritize their needs, empower themselves to recognize how addiction impacts them, and reject taking responsibility for their family member’s substance use disorder.^21^ To foster self-care, families must be provided with education to understand how living with substance use disorder affects them. Given the identified gap in resources and services for families affected by addiction, we decided to focus our grant application on developing an intervention for families affected by addiction in this region. This paper, therefore, addresses how we refined the ideas generated from the community knowledge-sharing event and sought a grant to gather more information about the needs and resources required to support families affected by addiction.

### Phase 2: Grant writing and award

In this phase, we developed a grant to gather more information about the needs and resources required to support families affected by addiction in this region. To make this work community-driven, we adopted a patient-oriented research approach to answer the following 2 research questions: (a) what are the experiences of living with a family member with problematic substance use and (b) what do families who are affected by addiction need to foster self-care? Patient-oriented research approaches are community-based, incorporating the perspectives and lived experiences of individuals, family members, and loved ones to inform the research process.^20^ Within the patient-oriented framework, patients and caregivers are considered to be experts. They can offer substantial knowledge regarding their health status, the preferred treatment modalities, and what they need to improve their quality of life and health outcomes.^22^

Involving various stakeholders as research partners is essential to addressing family members’ needs and preferences while developing new knowledge.^22^ Patients, caregivers, family advisors, and laypeople thus shared their exceptional wealth of knowledge, which is usually underused or ignored in traditional research.^23^ In addition to having community members with lived experiences as part of the research team, health care providers and decision-makers (managers in the health region and service agencies in the city) were represented in the research team. This composition increases the uptake of research findings to inform practices and policies.^24^ As a team, we proceeded to write a grant proposal entitled “Exploring the needs for and developing interventions for families affected by addiction in Prince Albert, Saskatchewan.” Community members, health care providers, and decision-makers provided input in the proposal’s design. This proposal was based on the previous community engagement process outcome reported elsewhere.^11^

In using community engagement, we hoped to understand the experiences of families affected by substance use disorder and addiction and develop support mechanisms to deal with the physical, emotional, and psychosocial impacts. We proposed using a community-based research methodology to guide the project as it would provide for active community involvement in both the process and the development of the outcome.^25^ In this manner, family members included in the research team were proactive in addressing addiction challenges in their families.

This research was co-funded by the Saskatchewan Health Research Foundation and Saskatchewan Centre for Patient-Oriented Research. Before the project began, research ethics approval was obtained from the University of Saskatchewan Behavioural Research and Ethics Review Board.

### Phase 3: Executing the study

In this phase, we carried out the project following the receipt of grant funding from the Saskatchewan Health Research Foundation and Saskatchewan Centre for Patient-Oriented Research. Individual interviews with participants who self-identified as affected by addiction (ie, had a loved one with problematic substance use) were the primary mode of data collection. Recruitment was done using word of mouth, email, and posters in strategic locations, such as the library and public notice boards, addiction treatment centers, detox units, and walk-in clinics.

Efforts were made to ensure that participants represented a diversity of ethnicities, ages, genders, and relationships with people with problematic substance use. Individual semi-structured in-depth interviews with participants focused on (a) their lived experiences caring for and living with a family member with problematic substance use; (b) the impact of addiction on their families; (c) their awareness of the need to seek professional help to deal with being a caregiver for a person with problematic substance use; (d) any unmet needs and resources needed for self-care and to support the person with problematic substance use. The research team developed the interview guide questions and was informed by literature review and community partner input. The following were broad questions asked: (a) demographic information; (b) childhood and adolescence experiences and past experiences with substance use; (c) loved ones affected by addiction; d) family involvement in addiction treatment of the loved one; (e) impact of addiction to the loved one and the family (socially, financially, physically, mentally, etc.); (f) the need for professional help to help address the substance use disorder; (g) resources might be needed for self-care and to support the family member with a substance use disorder.

Twenty-one participants participated in the interviews: 5 men and 16 women whose ages ranged from 27 to 72. Ten participants reported having siblings with substance use disorders, 5 had dependents with substance use disorders, 1 had a spouse with a substance use disorder, and 6 had parents with substance use disorders. The interviews, which lasted about 30 minutes, were recorded using a digital voice recorder and were transcribed verbatim.

Community partners and patients’ family members actively participated in preliminary data analysis, that is, developing a coding framework. In this phase, open coding of 3 rich interviews was undertaken and involved researchers, community members, and patients’ family advisors. The research team read 2 rich interviews and noted the meaning they derived from these interviews. Patterns and similarities emerging from the transcripts were identified and categorized as nodes. Further refinements resulted in a coding framework to analyze the remainder of the interviews using NVIVO 12 software for qualitative data management. A preliminary PowerPoint presentation comprising the study’s main findings was developed on the following themes: (a) impact of addiction on families (physical, mental, economic, and social); (b) factors shaping family experiences; and (c) self-care strategies for families affected by addiction.

### Phase 4: Knowledge translation activities

This phase describes the process and the outcome of sharing the study findings described in phase 3. Presenting the study’s findings to the community allowed us to inform the public about the participants’ understanding and perspective on the impacts of substance use on them. It was also a way of being accountable to the community and receiving further direction on the next steps. About 48 participants, comprising health care providers, social service providers, law enforcement officials, and community representatives, attended the event. The meeting began with a prayer from an Indigenous Elder, followed by a project recap. Study findings were presented, followed by a reflective exercise on issues that resonated with participants from the study findings. A summary of the issues arising from the discussion is categorized into the personal, community, and systemic issues and are summarized in Tables 1 to 3.

**Table 1.** Summary of participants’ reflection on the study findings.

Personal	Community	System
- Limited understanding of how addiction affects families - Inadequate information on how addiction is stressful to families - Families feel isolated and unsupported - The impact of addiction on families is invisible	- The community needs to know about how addiction is affecting families - “Powerful” excerpts on the effects of addiction on families can create communal awareness - Addiction stigma reduction in the community - Communal messaging that addiction affects us all - Individuals with substance use disorder and the effects on the family also effects the workplace	- Fragmented addiction services - Addiction services providers working in isolation - Inadequate addiction treatment - Lack of support services for families affected by addiction - Inadequate detox and treatment services - Outpatient services for people with problematic substance use - Better publicity of existing services - workplaces are importantly placed in the community to identify and support individuals with substance use disorder and their families

**Table 2.** Summary of the brainstorming session on how families can be best supported to mitigate the impact of addictions.

Action by the system	Support for the families
Education for families and community about addiction and how it impacts the family, including the intergenerational impact	Create a space for families affected by addiction to connect
Develop a family resource center and peer support structure	Promote community connections
Make addiction services inclusive, family-oriented, and family-focused	Promote workplace and community awareness of how addiction is affecting families to reduce stigma, trauma, and isolation
Better integration and coordination of services	Provide continuity and lessen support gaps
Address long waitlist	Increasing awareness of the existing services and support
Orient addiction services to include family recovery	Empower families to break the silence, take advocacy roles, challenge stigma, and have choices and autonomy
Act on provincial mental health strategy	Messaging—addiction is a family disease
More resources—shelters, long treatment cycles, counselling	Support for children affected by addiction
Provide education on family systems and how a healthy family function	Messaging—each family member has an important role to play to keep the family system working

**Table 3.** If addiction was addressed as a family illness, what changes need to happen in prevention, treatment, and rehabilitation programs?.

Prevention	Treatment	Rehabilitation/recovery
Educate children and youth on the impact of substances	Treat concurrent disorders and underlying issues	Give hope
Provide more information on addiction to the families and the public	Family-based addiction treatment	Address isolation
Promote healthy choices and healthy lifestyles	Holistic addiction treatment—physical, emotional, spiritual, and psychological	Harm reduction to keep hopes alive
Support positive parenting skills	Enact legislation	Create a social master plan for mental health and addiction
Addiction stigma reduction	Reduce wait times	Create a safe space for families affected by addiction to tell their stories
Focus on school health and school resources	Increase service	Give families affected by addiction a voice and a face
Early interventions for families to stay together	Consistent case-management approach	Foster support groups
Education for parents on addiction	Follow-up after crisis intervention	Address overt racism and social determinants of health
Train teachers to support students and families with addiction	Remove barriers to accessing care	Use non-stigmatizing language that is non-biased and non-blaming
Stigma reduction—addiction is a health and not a criminal issue	Promote continuum of care	Promote open community conversations about how addiction is affecting families and communities
Use social media to support families in rural communities	Actively involve male figures in addiction treatment	

Speakers were invited to share at this meeting. Mom Stop the Harm presented “Engaging with bereaved parent advocates as partners in substance use research and drug policy reform,” which summarized study findings on the advocacy work of bereaved mothers from opioid overdoses. Social workers who work with families at the Centre for Addiction and Mental Health presented methods for supporting families affected by addiction. A local advocate affected by addiction presented “Addiction crisis: Can we change the conversation to bring about recovery? Comments from a family perspective’s grassroots action.” She shared her journey, which entailed supporting a dependent through recovery, and her activism across the province.

Following these presentations, 2 brainstorming sessions titled “Family resource programming: What is needed?” and “Existing support systems and way forward: Guiding questions?” were held. Their outcomes are summarized in Table 2. After that, attendees in groups of 6 were asked to reflect on 2 questions: (a) How can families affected by addiction be best supported? and (b) If addiction was to be viewed as a family illness, what changes need to happen in prevention, treatment, and rehabilitation programs? The ideas generated were grouped by actions needed by the system and support required for the families, which were to be used in the intervention development.

Following the knowledge-sharing event, 2 working groups, composed of health care providers, social service providers, educators, researchers, and counsellors, were formed to develop interventions for families affected by substance use disorder and addiction. Information derived from the interviews and the knowledge-sharing event guided the working groups. During the first consultative meeting, educating and supporting families affected by addiction was considered a paramount intervention. Therefore, 2 working groups were tasked with creating psychoeducation videos using data from the research findings and with organizing an addiction awareness or recovery day. Due to COVID-19 restrictions, the focus of the intervention changed from developing video education tools to developing a toolkit. The process of creating and evaluating the toolkit will be reported in an outcome paper.

## Discussion

This research process was informed by the principles of community-based approaches described by Nilsen.^26^ These include having a community focus in this project: the community acted both as a target and catalyst for change. It also meant having active community member participation and intersectoral collaboration. In this case, we sought community members’ participation to better understand the problem and to incorporate a local perspective. We also involved representatives from the school division, community agencies, the health region and people with lived experiences as co-investigators in the grant. Finally, we had a long-term view. We intended to inject new perspectives on who is affected by addiction, and as such, open new lines of inquiry that can be pursued for years to come.

The community-based research approach was adopted to identify the needs of the research project. In both the knowledge exchange and the main study, unresolved grief, mental health challenges, and trauma associated with caring for family members with substance use disorders were significant recurring themes. Further, the lack of knowledge about how substance use affects families and the lack of support available for families was evident. Throughout this project, stakeholders’ and participants’ lived experiences underscored the extent of problematic substance use in the region. These findings validate previous reports and survey data regarding problematic substance use among residents of Prince Albert and the family in general.^1,13,14,16,20^

It is evident that family members’ experiences of living with loved ones affected by substance use disorders are under-researched, and their needs for social support continue to be overlooked.^13,14^ Using a stress-strain-coping-support model that focuses on family members’ situations and challenges has helped change the state of affairs regarding the impact of substance use on families.^14^ However, a significant observation was that few male caregivers were involved in the study or our community engagement events. We speculate that cultural and gender reasons may prevent men from participating in the study. Lopez-Anuarbe and Kohli suggest that, in general, the burden that men experience is underreported because they may be less accepting of their negative feelings, they may be uncomfortable sharing emotions, or they may be less in tune with how to process their emotions.^27^

Collaboration with stakeholders who provide addiction services has been essential in identifying the research questions, deepening our understanding of the impact of substance use on families, and identifying interventions to support families’ self-care. These collaborations have highlighted the need for community involvement in addressing substance use and addiction in the Prince Albert region. This evidence corroborated the findings of several studies, including systematic reviews, that showed a need to change, expand, and connect the focus of addiction programming to communal and family involvement, as these profoundly influence the recovery process of people with substance use disorders.^28-30^

The researchers, community members, health care providers, and stakeholders found ways to collectively identify culturally safe approaches to address substance use while empowering the community to be actively involved.^11,31,32^ Collaborative research with community members creates an environment that fosters relationships and increases the bidirectional connections between academics and community partners that can help overcome social challenges affecting communities.^33^

Throughout the different phases of the community-based participatory research process implemented in this project, we identified community priorities that would not have been obvious given the overemphasis on the person living with addiction. By involving people with lived experiences as co-investigators in the grant application and all of the study phases, the process and the outcome gained legitimacy that would otherwise have been missing in their absence. Inviting the community to be part of the knowledge exchange event and knowledge translation events refined the focus of the grant application and resource development respectively. Although it takes a lot of planning to involve the community in this manner, the community members who participated felt honoured that their voices were validated and used to share in the research process.

This work has increased awareness that addiction is a chronic illness with a significant impact on families. We believe that there will be ongoing conversations on substance use, recovery, and treatment that targets families, which should focus on (1) how addiction services should be structured to include families, (2) what support for families affected by addiction should look like, and (3) how to empower families to recover. Reorienting the focus of addiction from an individual to a family perspective can help address associated stigma and shame due to the scope of those affected by the issue. Moreover, as a result of this work, we believe that examining substance use disorder and its impact on families and communities in this holistic and integrated manner is the surest way to begin the recovery journey for the family. Community education will be paramount to understanding the complexity of substance use disorder as a chronic illness that also impacts the family. Thus, families can receive the support they need to recover.

This process has emboldened the research team to continuously involve the community in the research process as their active participation fostered community ownership of the process and the outcome. Identifying which community partner is required for a particular stage of the research process is critical. Some community partners were actively involved in the knowledge-sharing event while others refined the research question, and were engaged in grant writing, data collection, analysis, and knowledge translation. This diversity of involvement was occasioned by the availability of partners, the need to avoid burdening them and to spread out the burden and responsibility of the research process.

Community partners are now actively developing resources to support families affected by SUD and addiction. In this regard, we recognize that involving community partners in developing intervention strategies and resources increases the credibility of the process and optimizes usability and uptake of the outcome.^34^ We also depended on the community partners in identifying and using culturally safe language to communicate the research findings and convey community experiences. Since we were mindful of the complex language that has been used in the past to foster stigma, we adopted the community partners’ input. This necessitated a change in language from “addiction” to “substance use disorder” to reflect the community members’ voices. Understanding the rules of social contagion and using recovery-based language can decrease the stigma associated with mental health, including substance use disorders.^35^

Using community partners’ knowledge and experience to shape the research process increased our understanding of addiction as a chronic illness. They understood the role of research and communicated the importance of experiential knowledge in the research process.^30,36,37^ Like in Mosavel and Sanders’ work, involving community partners in our research process allowed for a critical reflection of their personal experiences and provided deeper insight into the problem based on how others experienced it.^30^

The involvement of community members as partners in the research process served to build their capacity to advocate for change in their community. Thus, their engagement in research and knowledge creation initiated change in the recovery process of substance use disorder and its impact on family life. Community engagement in research can produce results essential to the circumstances of the communities and their inhabitants’ lives.^33,38^ Community members’ unique experiences direct the research process. Having community members as partners enhances social justice by giving voice to family caregivers and service providers and catalyzes community empowerment and ownership of research findings and dissemination and intervention development.^31,37^

Despite support groups, such as Al-Anon, being in the community, the participants consistently expressed the need for community-driven interventions for families affected by substance use. The anonymity that characterizes the Al-Anon family program may have contributed to families being unaware of how and when it operates or its benefits. Even though participation in Alcoholic Anonymous (AA) and Al-Anon programs are open to everyone, studies have shown that their roots in spirituality and religion create some limitations related to participants’ diversity and age variations; for example, few young people participate because many, particularly young women, are often perceived as being too young to be alcoholics.^39^ Also, anonymity may be hard for people in small communities or areas. In addition, the absence of a program inventory means that the potential participants are unaware of their existence, which may lead to poor use. Much research indicates the benefits of joining support groups: they can help participants improve their self-esteem and self-confidence, empower themselves, share coping strategies, and provide mutual support, practical information, and reassurance about the commonality of their experiences.^40,41^

## Conclusion

Involving community partners in identifying and developing intervention strategies and resources is a rewarding experience that requires continuous engagement and commitment.^33^ The research team needs to negotiate power dynamics between researchers and community members on the identification of priorities and provide a way of accommodating divergent views among different partners. A successful collaboration between the research team and the community partners deepens the understanding of the issues that need to be prioritized and builds honesty, trust, and community buy-in. Regular meetings between the research team and the community partners helped clarify overt and covert substance use and addiction nuances expressed in the community gatherings and interviews. These meetings also helped validate observations regarding the research direction, including choices derived from the data on the intervention development.

This approach to identifying needs and developing interventions for families affected by substance use is time intensive. However, it was gratifying to witness the depth of information and the extent of community ownership throughout this process. We envision that a deeper understanding of the impact of substance use on families will lead to broader conversations regarding how families can be integrated into prevention, treatment, and rehabilitation services for substance use disorders and addictions. We also hope it will help to reduce the stigma that hinders the transparency needed to facilitate recovery. Through this work, we hope that thoughtful consideration of mainstreaming interventions for families affected by substance use disorders and addictions within the health care system will be integrated into the continuum of care.

We believe this work can be replicated in other settings that seek to actively involve the community in priority identification, grant writing, study implementation, knowledge translation, and intervention development. A couple of lessons that we have learned through this process are as follows:

1. To sustain community enthusiasm throughout the project, the research team and the community must identify a contemporary issue that is of major concern.
2. Investing in the community is a prerequisite, so they should be offered training on research modules, invited to professional conferences, and offered opportunities to co-author manuscripts.
3. The community engagement process takes time, and as such, there is a likely high turnover of community partners available to support the project in different phases.
4. To keep the community engaged, community partners should be regularly updated on the progress of the projects face to face or virtually.
